# Maternal peripheral blood telomere length and preterm birth in African American women: a pilot study

**DOI:** 10.1007/s00404-024-07681-1

**Published:** 2024-08-14

**Authors:** Weiyi Huang, Gang Han, Brandie DePaoli Taylor, Gabriel Neal, Kelli Kochan, Robin L. Page

**Affiliations:** 1 Department of Epidemiology and Biostatistics, Texas A &M University, College Station, TX USA; 2 Division of Basic Science and Translational Research, Department of Obstetrics and Gynecology, University of Texas Medical Branch, Galveston, TX USA; 3 Department of Primary Care and Rural Medicine, Texas A &M School of Medicine, Bryan, TX USA; 4 Institute for Genome Science and Society, Texas A &M University, College Station, TX USA; 5 School of Nursing, Texas A &M University, College Station, TX USA

**Keywords:** Preterm birth, Telomere length, African American

## Abstract

**Purpose:**

This study aimed to explore the association between preterm birth and telomere length of maternal peripheral blood in African American women.

**Methods:**

78 African American women were recruited for this study between 2018 and 2023 from 2 prenatal clinics in central and east Texas. Participants provided blood samples and completed clinic questionnaires, with clinical data collected from their post-delivery medical records. Telomere length was measured using monochrome multiplex quantitative real-time polymerase chain reaction. Linear regression and multinomial logistic regression were used to analyze the association between telomere length and gestational length. Kruskal–Wallis’s test and Fisher’s exact test were used to compare preterm birth, early-term birth and full-term birth by telomere length, social-demographic characteristics, stress and discrimination.

**Results:**

The rates of preterm birth was higher in pregnant women with shorter telomeres. After adjusting for confounders, for every 10-units increase in the relative telomere-to-single-copy gene (*T*/*S*) ratio, gestational days increased by 1.090 days (90% CI 0.182, 1.997), and for every 10-units decrease in the *T*/*S* ratio, the odds of preterm birth was 2.664 (90% CI 1.064, 6.673) times greater than the odds of full-term birth. No statistically significant associations were observed between stress, discrimination, and either preterm birth or telomere length.

**Conclusions:**

Maternal peripheral blood telomere shortening is associated with preterm birth, providing support to further explore the clinical utility of maternal telomere testing for prediction and early intervention of preterm birth and the study of biological mechanisms of spontaneous preterm birth.

## What does this study add to the clinical work

**Table:** 

This study suggests that telomere length in maternal peripheral blood holds promise as a new biomarker, which is crucial for devising preventive strategies and improving maternal and infant health outcomes.	

## Introduction

Preterm birth, with rates ranging from 5 to 18% worldwide, is a significant public health issue and a leading cause of infant mortality and morbidity  [1]. African American/Black women are disproportionately affected by preterm birth, with the disparity in preterm birth rates serving as a major factor contributing to racial disparities in infant mortality rates  [2]. The underlying causes of this disparity are multifactorial, including socioeconomic status, psychosocial stress, as well as genetic and biological factors  [3].

Stressful life events experienced during pregnancy are associated with preterm birth  [4]. When faced with stress, the hypothalamus releases corticotropin-releasing hormone (CRH), which stimulates the anterior pituitary gland to release adrenocorticotropic hormone (ACTH). ACTH then stimulates the adrenal cortex to release cortisol, a stress hormone. This series of reactions constitutes the HPA axis (hypothalamic–pituitary–adrenal axis), which plays a crucial role in regulating the body’s response to stress. Chronic stress or HPA axis dysregulation can lead to elevated cortisol levels, which are associated with an increased risk of preterm birth, offering a biological explanation for the disparity in preterm birth rates between African American and white women due to stress  [5].

Telomere, a DNA-protein compound structure at the end of a chromosome, has been recognized not only as a biomarker but also as a mediator by which chronic psychosocial stress contributes to the development of diseases  [6]. Stress activates the hypothalamic–pituitary–adrenal (HPA) axis, leading to a surge of glucocorticoids in the bloodstream, which in turn increases mitochondrial activity, generating reactive oxygen species (ROS). ROS damages telomeres and inhibits telomerase activity, resulting in telomere shortening and dysfunction. Additionally, glucocorticoids promote inflammation, which can lead to viral chronic infections characterized by shortened and dysfunctional telomeres, accelerating telomere loss. These biological mechanisms explain the relationship between telomere shortening and stress  [6].

Although these studies have indicated a relationship between telomere shortening and stress, as well as a link between stress and preterm birth, along with the potential underlying biological mechanisms, the association between telomere length and preterm birth remains unclear. One hypothesis suggests that when telomeres in gestational tissues (i.e. the placenta and fetal membranes) decrease to a critical level, it triggers a series of signaling events, leading to the activation of proinflammatory, endocrine, and uterotonic phenomena, then resulting in cervical ripening, phasic myometrial contractions, and rupture of the fetal membranes, ultimately culminating in spontaneous delivery  [7]. African American women may face various risk factors for preterm birth, including psychological stress, low socioeconomic status, racial discrimination, smoking, and low educational attainment, some of which are associated with an increased rate of preterm birth  [8]. This study aims to explore the relationship between telomere length and preterm birth, as well as their associations with stress, discrimination, socioeconomic status, smoking, and other risk factors, thereby providing evidence to open possibilities for new methods of prevention or reduction of preterm birth.

## Methods

### Study population and data collection

The study was an exploratory, prospective pilot study that recruited 78 women through convenience sampling between September 2018 and February 2023. These women were between 17 weeks 1 day and 36 weeks 4 days pregnant and were existing patients at two prenatal clinics in central and east Texas. They were aged between 18 and 40 years and self-identified as Black or African American. Exclusion criteria included in vitro fertilization, multiple gestation, planning to transfer care before delivery, and inability to complete surveys in English. The study received approval from the university’s institutional review board (IRB2018-0407D), and all participants provided written informed consent. Participants completed a paper questionnaire and provided a tube of whole blood for telomere analysis. The final sample size was 75 cases since three participants’ qPCR results were excluded due to lab error. Among enrolled participants, some were missing data on gestational week, telomere length, perceived stress levels, and socioeconomic characteristics. Given the study’s limited sample size, we did not exclude participants with missing data from the analysis.

### Measures

#### Preterm birth/early-term birth/full-term birth

The gestational length was determined through retrospective chart review conducted after the participants gave birth. Gestational lengths were categorized as follows: preterm: < 37 weeks; early term: 37 weeks 0 days through 38 weeks 6 days; full term: 39 weeks 0 days through 40 weeks 6 days (American College of Obstetricians & Gynecologists, 2012).

#### Telomere length

Initially, whole blood was centrifuged within 24 h of collection to isolate the plasma fraction, with the buffy coat layer (comprising white blood cells) collected and preserved at − 80 °C until all participants were enrolled. Subsequently, samples were transported to the Institution’s core genomics facility, stored at − 80 °C until white blood cell isolation, DNA extraction, and telomere length analysis commenced. DNA extraction from the buffy coats involved washing white blood cell pellets to eliminate circulating cell-free DNA, ensuring telomere length analysis reflects intact maternal cells. Telomere length assessment employed monochrome multiplex quantitative real-time polymerase chain reaction (qPCR) following Cawthon’s delineation  [9]. Each sample was triplicate-amplified on two 384-well plates alongside a standard curve and negative control, with amplification data analyzed using CFX Maestro software to derive quantity data. The relative telomere-to-single-copy gene (*T*/*S*) ratio was calculated as per Cawthon’s method  [9]. Samples exhibiting intra-plate coefficient of variation (CV%) > 10% among triplicates or inter-plate CV% > 10% between the two plates were re-run within 2 days, with fresh aliquots of DNA utilized if further reruns were warranted.

#### Perceived stress

The perceived stress was measured using the perceived stress scale PSS-10  [10], which is a commonly used psychological measurement tool designed to assess an individual’s perceived level of stress over the past month. It consists of ten statements that cover various aspects of stress related to events and feelings in life, which has two subscales: perceived helplessness scale and lack of self-efficacy scale. Perceived helplessness scale measures individuals’ feelings of lacking control over their situation or emotions, with participants self-reporting their level of agreement on a scale from 0 to 4 (where 0 = never and 4 = very often). Lack of self-efficacy scale assesses individuals’ ability to deal with problems, with participants reporting scores from 0 to 4 (where 0 = very often and 4 = never). Participants whose total scores for all these questions were below the 25th percentile were categorized as having low perceived stress, while those whose total scores were equal to or above the 25th percentile were categorized as having moderate or high stress.

#### Discrimination experience

Participants were questioned about their encounters with discrimination or instances where they were hindered or made to feel inferior due to their race, ethnicity, or color. We used the validated “Experiences of Discrimination” scale by Krieger et al. [11]. This inquiry encompassed nine distinct scenarios: experiences at school, challenges in securing employment, workplace discrimination, difficulties in obtaining housing, encounters within the healthcare system, instances of receiving service in commercial establishments, obstacles in accessing financial services such as credit, loans, or mortgages, interactions in public spaces or on the streets, and encounters with law enforcement or the judicial system. Each scenario was assessed using the following scale: None; Yes, once; Yes, 2–3 times; and Yes, 4 or more times. Participants who reported no experiences or experiences only once were categorized as having a low level of discrimination experiences, while those who reported two or more occurrences in at least one of the nine scenarios were categorized as having a high level of discrimination experiences.

### Statistical analyses

The descriptive statistics include sociodemographic characteristics (i.e. age, marital status, education, employment status, household income, smoking status), perceived stress, discrimination experience, telomere length, and history of preterm birth. Categorical variables were reported with frequency and percentage, while continuous variables were reported with median and interquartile range (IQR). Kruskal–Wallis’s test and Fisher’s exact test were used to compare the preterm birth group, early-term group, and full-term group by telomere length, social-demographic characteristics, self-perceived stress, discrimination experience, smoking and history of preterm birth. Gestational length was treated as a multinomial variable, and we also calculated the median *T*/*S* ratio for the full sample for comparative analysis between gestational length and the overall median telomere length (lower than vs. higher than the median *T*/*S* ratio). Linear regression and multinomial logistic regression were used to analyze the association between telomere length and gestational length. The adjusted models included age, smoking behavior, preterm birth history, perceived stress, and discrimination experience as confounders. A *p* value was considered significant if it was less than 0.05, and marginally significant if it was equal to or greater than 0.05 but less than 0.1. Statistical analysis was performed using SAS software version 9.4 (SAS Institute, Cary, NC, USA).

## Results

Our findings revealed a 10.81% prevalence of preterm birth among our sample of African American women. Of these African American women, 28% were primiparous women and 72% were multiparous women. The median age of mothers in the preterm birth group was the highest at 29.5 years, whereas the median ages for early-term and full-term pregnancies were both at 24.0 years. The preterm birth group had lower median of the *T*/*S* ratio at 195.6, while early-term and full-term pregnancy groups had higher *T*/*S* ratio median, at 209.1 and 216.6, respectively. Kruskal–Wallis’s Test indicated that these median differences were statistically significant (*p* = 0.0059, Table 1). We categorized samples based on the *T*/*S* ratio median, as “lower than median” and “higher than median”. It is noteworthy that the proportion of “lower than median” was highest in the preterm birth group at 87.50%, contrasting with lower proportions in the early-term and full-term groups, at 39.39% and 33.33% respectively. Fisher’s exact test showed that these proportional differences were statistically significant (*p* = 0.0347, Table 1).

In addition, smoking behaviors and preterm birth history differed significantly among women in the preterm birth, early-term birth, and full-term birth groups. The preterm birth group had the largest proportion of smoking women (62.50%), substantially higher than the rates in the early-term birth and full-term birth groups at 18.18% and 9.09% respectively ($P=0.0073$, Table 1). And, the prevalence of preterm birth was substantially higher among women who smoked, compared to nonsmokers (35.71% vs. 5.00%, $P=0.0073$, Table 2). Similarly, in the preterm birth group, half of the women had a history of preterm births, which was significantly more than the other two groups (3.03% and 9.09%, respectively, $p=0.0060$, Table 1), and the prevalence of preterm birth was substantially higher among women who had a history of preterm birth, compared to those who didn’t (50.00% vs. 6.06%, $P=0.0060$, Table 2). Univariate analysis (Tables 1, 2) showed that there was no statistically significant difference in telomere length or preterm birth rate between multiparous and primiparous women. While not statistically significant, the preterm birth group had the largest number of women reporting high stress and incidents of discrimination (75.00% and 25.00%, respectively), and the prevalence of preterm birth was higher among women with higher stress and discrimination levels compared to those with low levels (Table 2). Marital status and household income were marginally significantly associated with gestational length ($P= 0.0954$ and $P= 0.0739$, respectively, Table 1). We also analyzed the association between covariates and telomere length. Although none of them reached statistical significance, women with *T*/*S* ratio lower than median tended to be older compared to those with *T*/*S* ratio higher than median (25.0 vs. 24.0 years of age), and women who reported higher perceived stress had a higher rate of “below *T*/*S* ratio median” to those with low perceived stress (52.08% vs. 33.33%). In addition, the women who reported higher incidences of discrimination (57.14% vs. 47.92%), who were smokers (75.00% vs. 44.00%), and who had the preterm birth history (57.14% vs. 49.09%) had a higher rate of “below *T*/*S* ratio median” (Table 2).

Furthermore, we evaluated the association between telomere length and gestational length. In the unadjusted model, every 10-units increase in the *T*/*S* ratio was associated with a change in gestational length of 2.046 days (90% CI 0.541, 3.550; Table 3). After adjusting for confounders, the association persisted but attenuated. The adjusted change in gestational length for every 10-units increase in the *T*/*S* ratio was 1.090 days (90% CI 0.182, 1.997; Table 3). These findings suggested that telomere length was associated with gestational length, with an increase in telomere length being associated with an increase in gestational length, even after accounting for confounders. Additionally, we analyzed the associations between categories of gestational length and telomere length. In the unadjusted model, for every 10-units decrease in the *T*/*S* ratio, the odds of preterm birth was 2.387 (90% CI 1.441, 3.955; Table 4) times greater than the odds of full-term birth, while the odds of early-term birth was not statistically significantly greater. After adjusting for confounders, for every 10-units decrease in the *T*/*S* ratio, the odds of preterm birth was 2.664 (90% CI 1.064, 6.673; Table 4) times greater than the odds of full-term birth, while the odds of early-term birth was not statistically significantly greater.

## Discussion

To the best of our knowledge, this is the first study to explore the association between maternal peripheral blood telomere length and preterm birth among African American pregnant women. In a previous prospective study, researchers collected placental tissue samples from the amnion, chorion, villi, and umbilical cord to measure telomere length, demonstrating the relationship between telomere length differences and racial disparities in preterm birth  [12]. Another study  [13] reached similar conclusions on fetal telomere shortening and preterm birth by assessing telomere length in fetal umbilical cord blood leukocytes. Our study, however, is the first to use maternal peripheral blood to validate this relationship. Our research demonstrated an association between telomere length in maternal peripheral blood and the odds of preterm birth. This relationship provides the potential for clinical application of maternal peripheral blood measurements to non-invasive prenatal prediction and intervene early in preterm birth.

Telomeres are highly conserved structures at the ends of chromosomes, which have been demonstrated as biomarkers of premature placental aging  [14], a condition related to preterm delivery. Pregnancy is considered a state of high oxidative stress  [15], where free radicals generated during oxidative stress attack the guanine nucleotide triplets within telomere repeat sequences, leading to telomere DNA breakage  [16]. These DNA fragments induce pro-inflammatory innate immune responses, subsequently stimulating endocrine signaling and activation of uterine contractions, resulting in labor, cervical ripening, phase-specific muscle contractions, and membrane rupture  [17]. A case–control study  [18] revealed that pregnant women with threatened preterm labor had elevated levels of serum oxidative stress, and oxidative stress is one of the pathophysiological mechanisms associated with preterm birth. Antioxidants can mitigate the effects of oxidative stress, and antioxidant vitamins have been shown to alleviate oxidative stress, thereby delaying or preventing telomere shortening  [19], while deficiency in antioxidant vitamins has been reported to be associated with spontaneous preterm birth with premature rupture of membranes  [20]. Oxidative stress, as a potential biological mechanism underlying preterm birth, is related to another high-risk factor of preterm delivery: smoking. Our study revealed that among women who had preterm birth, the proportion of smokers was higher than non-smokers. Also, the prevalence of preterm birth is higher among women who were smokers compared to those who were non-smokers, consistent with previous research findings  [21, 22]. Pregnant women who smoke exhibit two to three times higher levels of cadmium in the placenta, and the increased oxidative stress induced by placental cadmium leads to endothelial cell damage in placental blood vessels, thereby contributing to preterm delivery  [23].

**Table 1:** Characteristics of African American women in preterm, early-term and full-term births

		Full-term $(n=33)$	Early term $(n=33)$	Pre-term $(n=8)$	*P* value
Age, Median [IQR]	24.0 [22.0, 29.0] $(n=75)$	24.0 [21.0, 28.0] $(n=33)$	24.0 [22.0, 28.0] $(n=33)$	29.5 [25.5, 34.0] $(n=8)$	0.1114
*Telomere length Median [IQR]*	212.5 [200.7, 226.7] $(n=63)$	216.6 [209.5, 226.9] $(n=30)$	209.1 [199.6, 226.2] $(n=24)$	195.6 [188.0, 201.3] $(n=8)$	0.0059
1. Less than median	31 (41.33%)	11 (33.33%)	13 (39.39%)	7 (87.50%)	0.0347
2. More than median	31 (41.33%)	18 (54.55%)	11 (33.33%)	1 (12.50%)	
Missing	13 (17.33%)	4 (12.12%)	9 (27.27%)	0 (0.00%)	
*Perceived stress*					0.5771
1. Low	10 (13.33%)	6 (18.18%)	5 (15.15%)	1 (12.50%)	
2. Moderate or high	59 (78.67%)	22 (66.67%)	27 (81.82%)	6 (75.00%)	
Missing	6 (8.00%)	5 (15.15%)	1 (3.03%)	1 (12.50%)	
*Discrimination*					1.0000
1. Low	58 (77.33%)	26 (78.79%)	25 (75.76%)	6 (75.00%)	
2. High	17 (22.67%)	7 (21.21%)	8 (24.24%)	2 (25.00%)	
*Marriage*					0.0954
1. Single/never married	59 (78.67%)	29 (87.88%)	24 (72.73%)	5 (62.50%)	
2. Married/domestic partnership	11 (14.67%)	1 (3.03%)	7 (21.21%)	3 (37.50%)	
3. Divorced/separated/widowed	4 (5.33%)	3 (9.09%)	1 (3.03%)	0 (0.00%)	
Missing	1 (1.33%)	0 (0.00%)	1 (3.03%)	0 (0.00%)	
*Education*					0.1079
1. High school or less	43 (57.33%)	24 (72.73%)	15 (45.45%)	4 (50.00%)	
2. More than high school	31 (41.33%)	9 (27.27%)	17 (51.52%)	4 (50.00%)	
Missing	1 (1.33%)	0 (0.00%)	1 (3.03%)	0 (0.00%)	
*Employment*					0.5828
1. Employed	37 (49.33%)	18 (54.55%)	14 (42.42%)	4 (50.00%)	
2. Unemployed	38 (50.67%)	15 (45.45%)	19 (57.58%)	4 (50.00%)	
*Household income*					0.0739
20,000 or less	54 (72.00%)	28 (84.85%)	21 (63.64%)	4 (50.00%)	
More than 20,000	21 (28.00%)	5 (15.15%)	12 (36.36%)	4 (50.00%)	
*Cigarette smoking*					0.0073
No	61 (81.33%)	30 (90.91%)	27 (81.82%)	3 (37.50%)	
Yes	14 (18.67%)	3 (9.09%)	6 (18.18%)	5 (62.50%)	
*History of Preterm birth*					0.0060
No	67 (89.33%)	30 (90.91%)	32 (96.97%)	4 (50.00%)	
Yes	8 (10.67%)	3 (9.09%)	1 (3.03%)	4 (50.00%)	
*Parity*					0.5920
Primiparous	21 (28.00%)	7 (21.21%)	12 (36.36%)	2 (25.00%)	
Multiparous	54 (72.00%)	26 (78.79%)	21 (63.64%)	6 (75.00%)

**Table 2:** Characteristics of African American women in relation to telomere length and gestational length

	By categories of gestational length				By telomere length		
	Full-term $(n=33)$	Early term $(n=33)$	Pre-term $(n=8)$	*P* value	*T*/*S* > median $(n=31)$	*T*/*S* < median $(n=31)$	*P* value
*Age, median [IQR]*	24.0 [21.0, 28.0] (*n* = 33)	24.0 [22.0, 28.0] (*n* = 33)	29.5 [25.5, 34.0] (*n* = 8)	0.1114	24.0 [22.0, 28.0] (*n* = 31)	25.0 [21.0, 29.0] (*n* = 31)	0.9662
*Perceived stress*				0.5771			0.5746
1. Low	6 (50.00%)	5 (41.67%)	1 (8.33%)		6 (66.67%)	3 (33.33%)	
2. Moderate or high	22 (40.00%)	27 (49.09%)	6 (10.91%)		23 (47.92%)	25 (52.08%)	
Missing	5 (71.43%)	1 (14.29%)	1 (14.29%)		2 (40.00%)	3 (60.00%)	
*Discrimination*				1.0000			0.9334
1. Low	26 (45.61%)	25 (43.86%)	6 (10.53%)		25 (52.08%)	23 (47.92%)	
2. High	7 (41.18%)	8 (47.06%)	2 (11.76%)		6 (42.86%)	8 (57.14%)	
*Cigarette smoking*				0.0073			0.1756
No	30 (50.00%)	27 (45.00%)	3 (5.00%)		28 (56.00%)	22 (44.00%)	
Yes	3 (21.43%)	6 (42.86%)	5 (35.71%)		3 (25.00%)	9 (75.00%)	
*History of Preterm birth*				0.0060			1.0000
No	30 (45.45%)	32 (48.48%)	4 (6.06%)		28 (50.91%)	27 (49.09%)	
Yes	3 (37.50%)	1 (12.50%)	4 (50.00%)		3 (42.86%)	4 (57.14%)	
*Parity*				0.5920			0.4324
Primiparous	7 (33.33%)	12 (57.14%)	2 (9.52%)		11 (57.89%)	8 (42.11%)	
Multiparous	26 (49.06%)	21 (39.62%)	6 (11.32%)		20 (46.51%)	23 (53.49%)

**Table 3:** Unadjusted and adjusted change in gestational length with 10-units increase in the *T*/*S* ratio

Covariate adjustment	Change in gestational length (days) (with 90% CI)	*p* value
Unadjusted	2.046 (0.541, 3.550)	0.0267
Adjusted^a^	1.090 (0.182, 1.997)	0.0496

^a^Model adjusted for age, smoking, preterm birth history, self-perceived stress, and discrimination experience

**Table 4:** Unadjusted and adjusted associations between categories of gestational length and 10-units decrease in the *T*/*S* ratio

	Early-term vs. full-term	*p* value	Preterm vs. full-term	*p* value
Unadjusted odds ratio (with 90% CI)	1.153 (0.918, 1.446)	0.3057	2.387 (1.441, 3.955)	0.0046
Adjusted odds ratio^a^ (with 90% CI)	1.085 (0.863, 1.365)	0.5558	2.664 (1.064, 6.673)	0.0790

^a^Model adjusted for age, smoking, preterm birth history, self-perceived stress, and discrimination experience

Compared to white women, African American women have a higher rate of preterm birth, which may be attributed to race-specific social, psychological, or environmental stressors  [24]. We investigated the relationship between gestational length and perceived stress and discrimination experience, finding that women with higher levels of self-perceived stress and more experiences of discrimination had higher rates of preterm delivery. However, this difference is minor, perhaps because our study only included a single ethnic group, thereby unable to detect significant variations. Although this result lacked statistical significance, the small sample size of this study influences the *p* value  [25]. Stress and discrimination experiences were biologically explained in relation to preterm birth through neuroendocrine, immune/inflammatory, and vascular processes  [26], and the increase in maternal stress levels was associated with telomere shortening in fetal umbilical cord blood  [27]. It is noteworthy that a significant proportion of African American women with preterm births in our study had a history of preterm birth, which may be explained by intergenerational preterm birth recurrence  [28]. Smid’s study  [28] suggested that this intergenerational effect was based on genetic or epigenetic phenomena, while telomere shortening was an epigenetic phenomenon modulated by life experiences. Among the socio-demographic factors, we observed a marginally significant association of preterm birth with household income and marital status. However, the high rate of preterm birth among African American women cannot be solely explained by social demographic factors  [3]. Our research findings indicated that even after adjusting for social and environmental exposures, i.e., age, smoking, self-perceived stress and discrimination experience, the association between telomere shortening and preterm delivery remained significant. This indicated that the impact of telomere shortening on preterm birth was independent of these social and environmental exposure factors, so there are potentially undiscovered biological mechanisms that contribute to the role of telomere in explaining preterm birth.

## Conclusions

This pilot study suggested an association between maternal blood telomere length and preterm birth, although residual and unreported confounding is a possibility. Despite our small sample size, we still observed a statistically significant association between them, providing evidence that further clinical and mechanistic studies are warranted. Future studies may determine if telomere length explains some of the racial disparities in pretrem birth rates. A multicenter and multiethnic study in the future may offer more insights into the mechanisms that explain racial disparities in preterm birth.

## Data Availability

Data were available from the the corresponding author upon reasonable request.
